# Complete Genome Sequence of Halomonas meridiana Strain Slthf1, Isolated from a Deep-Sea Thermal Vent

**DOI:** 10.1128/MRA.00292-20

**Published:** 2020-04-16

**Authors:** Yuka Takahashi, Haruno Takahashi, Josephine Galipon, Kazuharu Arakawa

**Affiliations:** a Institute for Advanced Biosciences, Keio University, Tsuruoka, Japan; b Systems Biology Program, Graduate School of Media and Governance, Keio University, Fujisawa, Japan; c Faculty of Environment and Information Studies, Keio University, Fujisawa, Japan; d Exploratory Research Center on Life and Living Systems, National Institutes of Natural Sciences, Myodaiji, Okazaki, Japan; Georgia Institute of Technology

## Abstract

Halomonas meridiana strain Slthf1 (ATCC BAA-801) is a Gram-negative bacterium that was isolated from a thermal vent in 1998. Here, we report the complete genome sequence of this strain, which has a 3.6-Mbp genome, containing 3,400 protein-coding sequences.

## ANNOUNCEMENT

*Halomonas* species are a group of Gram-negative halophiles belonging to the phylum *Proteobacteria*, order *Oceanospirillales*, and family *Halomonadaceae*. Within this genus, Halomonas meridiana has been reported to inhabit high-salt lakes in Antarctica (1). The number of halophilic bacteria known to live in such environments is limited, probably due to the great seasonal variation in the physicochemical conditions of Antarctic high-salinity lakes. Thus, determining the genomic sequences of *H. meridiana* and its relatives is expected to contribute to our understanding of these ecological adaptations. Here, we report the complete genome sequence of *H. meridiana* strain Slthf1 (ATCC BAA-801), isolated from the vicinity of a Pacific hydrothermal vent by Kaye et al. (2).

*H. meridiana* strain Slthf1 was obtained directly from J. Z. Kaye. A single colony was cultured overnight at 37°C using SW10 medium (3). The genomic DNA was extracted and purified using a Genome-tip 20/G kit (Qiagen) according to the manufacturer’s protocol. A long-read sequencing library was prepared using a rapid barcoding sequencing kit (product no. SQK-RBK004; Oxford Nanopore Technologies) and sequenced using a FLO-MIN106 flow cell on a GridION device (Oxford Nanopore Technologies). Illumina sequencing was performed for error correction using the HyperPlus kit (Kapa Biosystems) for library preparation and a NextSeq 500 sequencer in 75-cycle high-output mode (Illumina). From a total of 71,261 long reads with an average read length of 12,993 bp, reads over 50 kbp (291 Mbp in total, for an estimated coverage of around 80×) were used for assembly with Canu version 1.8 (4). The resulting single contig was circularized by manually deleting the overlapping ends. Error correction was performed using Illumina reads, by mapping all 49.3 million reads without filtering with BWA version 0.7.11 (5), indexing with SAMtools version 1.9 (6), and correcting with Pilon version 1.23 (7). Assembly completeness was evaluated using Benchmarking Universal Single-Copy Orthologs (BUSCO) version 1 (8) and the gVolante server (9). Six rounds of Pilon error correction increased the BUSCO score from 77.5% to 100%. Genes were annotated using the DDBJ Fast Annotation and Submission Tool (DFAST) pipeline (10). The final genome size of this strain is 3,591,816 bp, with a G+C content of 56.8%. The genome was predicted to contain 3,400 protein coding sequences (CDSs), 18 rRNAs, and 64 tRNAs. Default parameters were used for all software unless otherwise noted.

Three cold shock proteins were identified from the genome of *H. meridiana* strain Slthf1 (ATCC BAA-801) according to the DFAST annotation. When the three amino acid sequences were searched for using BLASTp in the NCBI nonredundant (nr) database (11), they showed high similarity to the Csp protein family (E value, 8e-34). The complete genome reported in this study will enable comparative analysis with closely related species to elucidate the mechanisms allowing halophilic microorganisms to thrive in cold environments.

### Data availability.

The chromosome sequence reported here was deposited in DDBJ under the accession no. AP022821, and the raw reads were deposited in the Sequence Read Archive (SRA) under BioProject no. PRJNA605950.
